# Workforce Development in Integrated Care: A Scoping Review

**DOI:** 10.5334/ijic.6004

**Published:** 2021-11-25

**Authors:** Frances Barraclough, Jennifer Smith-Merry, Viktoria Stein, Sabrina Pit

**Affiliations:** 1 University Centre for Rural Health, University of Sydney, AU; 2 University of Sydney, AU; 3 Leiden University Medical Centre, Department of Public Health and Primary Care, AU

**Keywords:** integrated care, health workforce, workforce development, scoping review

## Abstract

**Introduction:**

Integrated care aims to improve access, quality and continuity of services for ageing populations and people experiencing chronic conditions. However, the health and social care workforce is ill equipped to address complex patient care needs due to working and training in silos. This paper describes the extent and nature of the evidence on workforce development in integrated care to inform future research, policy and practice.

**Methods:**

A scoping review was conducted to map the key concepts and available evidence related to workforce development in integrated care.

**Results:**

Sixty-two published studies were included. Essential skills and competencies included enhancing workforce understanding across the health and social care systems, developing a deeper relationship with and empowering patients and their carers, understanding community needs, patient-centeredness, health promotion, disease prevention, interprofessional training and teamwork and being a role model. The paper also identified training models and barriers/challenges to workforce development in integrated care.

**Discussion and Conclusion:**

Good-quality research on workforce development in integrated care is scarce. The literature overwhelmingly recognises that integrated care training and workforce development is required, and emerging frameworks and competencies have been developed. More knowledge is needed to implement and evaluate these frameworks, including the broader health and social care workforces within a global context. Further research needs to focus on the most effective methods for implementing these competencies.

## Introduction

Internationally, governments have committed to integrated health systems to improve access, quality and continuity of services for our increasingly ageing population and people experiencing chronic disease [1,2,3]. Patients with ongoing health problems need continuous care and treatment across settings and providers [Pruitt & Epping-Jordan 2005, as cited in 1]. Growing evidence supports an integrated approach between healthcare and other sectors, emphasising a person-centred, preventative and community-based approach rather than disease-based and institution-focused care [1]. An integrated approach requires workers from several sectors to collaborate with patients, carers and each other to develop personalised treatment plans that reflect patient and family needs, preferences and community resource and service availability [4,5,6, Pruitt and Epping—Jordan 2005, as cited in 1]. Moreover, in 2016, the World Health Organization (WHO) endorsed a global framework on integrated people-centred health services. The framework imposed five interdependent strategies: empowering and engaging people and communities, strengthening governance and accountability, reorienting care models, coordinating services within and across sectors and creating an enabling environment [7].

Before evaluating the research into workforce development in integrated care, some definitions are needed. The WHO defines integrated care as:

The management and delivery of health services such that people receive a continuum of health promotion, health protection and disease prevention services, as well as diagnosis, treatment, long-term care, rehabilitation, and palliative care services through the different levels and sites of care within the health system and according to their needs [7].

By ‘health and social care workforce’, we mean ‘the different kinds of clinical and non-clinical staff responsible for public and primary health interventions’ [7].

Barriers to implementing integrated services are well-described in the literature. They include a lack of clear, systematic understanding of integrated care among key stakeholders [8] and a lack of standardised, validated tools and indicators to measure integration [3]. Previous research also describes workforce challenges, including an existing, entrenched workforce culture, limited opportunities for cooperation and communication, interprofessional education, resistance to share care, high costs, staff skills and information technology systems [9,10,11].

Similar barriers exist in workforce development. For example, training curricula for healthcare workers do not promote experience and skills in community and integrated care settings [12,13]. Students learn primary care principles in many training programmes but are then placed in clinical environments where it is difficult to implement and practice those principles [14]. Limited access to experts inhibits the scaling up of existing competencies and curricula [4]. Faculty expertise, financial, organisational and logistical factors are other recognised barriers to implementing integrated curricula into healthcare workers’ training [15,10,11]. Training still emphasises the diagnosis and treatment of acute diseases, fragmented, outdated and static curricula, and education and practice that focus on component elements of an issue or disease [16].

Professionals need to manage health and care rather than disease and cure. To work in teams across professions and sectors, they need to acquire a non-traditional set of knowledge, skills and attitudes [8]. Although the literature contains many models and examples of integrated care systems in different settings and populations, there is insufficient discussion of how the health and social care workforce have prepared and trained to work within these settings [9,11]. Therefore, this article systematically reviews the literature on workforce development, including characteristics, models, key competencies, barriers, challenges and global recommendations. The review is the first component of a broader project to develop an international education-focused framework for health and social care professional education.

## Methods

A scoping review was selected to map the key concepts and available evidence of how education, training and workforce development in integrated care systems have been implemented and represented in the literature. The goal is to synthesise the research into education and training by mapping or articulating current knowledge about these critical concepts derived from various study designs. A scoping review is particularly relevant in this field, as emerging evidence makes it challenging to undertake systematic reviews [17]: and scoping reviews allow for a broader range of study types to be included [18]. As a result, the method allows for knowledge strengths and gaps to be identified and set within policy and practice contexts.

The research questions, protocol, scoping review process and inclusion criteria for the search strategy were developed in consultation with a group of experts with knowledge of integrated care and working in health and social science. These experts assisted with the initial review of full articles for inclusion and a descriptive numerical summary of the evidence. The scoping review followed Levac’s [17] recommendations, Arksey and O’Malley’s [18] five-stage protocol and the Joanna Briggs Institute Preferred Reporting Items for Systematic Reviews and Meta-analyses Extension for Scoping Reviews checklist [19]. The five stages of this scoping review consisted of the following: (1) defining the research questions and purpose, (2) identifying relevant studies, (3) study selection, (4) data charting and (5) the collation, summarising and reporting of results [18].

### Stage 1: Defining the Research Questions and Purpose

A broad question, key concepts, target audience and intended outcome, were defined for the study (see ***Table 1***). Although an integrated workforce includes many stakeholders (such as government, social support sectors, including education and housing and individuals’ families and communities), the scoping review focuses on the training of health and social care workers.

**Table 1:** Scoping Review Methods.


SCOPING REVIEW STAGE	METHODS

(1) Defined the research questions and purpose	The following research question was developed: What is known from the existing literature about workforce development in integrated care? The scoping review focused on two concepts: (1) integrated care and (2) workforce development Target audience for review: healthcare workers Intended outcomes: a thematic framework that represents the key concepts and contexts for education and training a list of the key future research priorities.

(2) Identified relevant studies	Search strategy: Initial limited searches were conducted in PubMed to identify relevant keywords and MeSH terms. This list of terms and MeSH synonyms was developed with reference to the two concepts and applied to CINAHL and Medline databases to test for relevance. Abstracts of potentially useful studies were read to identify any other relevant search terms. The search also included input from a senior health science librarian. A similar search strategy was used for all databases. Databases searched: Medline, CINAHL, EMBASE, ERIC (education, policy and theory), Cochrane, Web of Science and Scopus Initial eligibility criteria: Articles written in English Articles published between 2013 and 2020 Refined inclusion and exclusion criteria: Articles were included if they described an educational model or framework and key elements or competencies in health workforce training, education and integrated care. Articles were excluded if they had a single disease focus, were conference abstracts, there was no full text available or were not in English.

(3) Selected studies	Article titles and abstracts were screened to ensure that they explicitly discussed health workforce training, education and integrated care. Full articles were then screened and pilot tested, and inclusion criteria refined until they were considered fit for purpose. Three authors developed and piloted a standardised full text table to calibrate and test the full text data extraction. One author extracted the data using the table, with two additional authors checking for completeness and independently screening at least 20% of full text articles [17].

(4) Charted the data	Extracted material included authors, year, title, country, journal, type of study (i.e., empirical/non-empirical) target workforce, skills and competencies, programme models, use of participants in the programme design, study recommendations and a summary of a perfect workforce.

(5) Collated, summarised and reported the results	


*Notes*: MeSH = Medical subject heading.

### Stage 2: Identifying Relevant Studies

**Figure 1:**
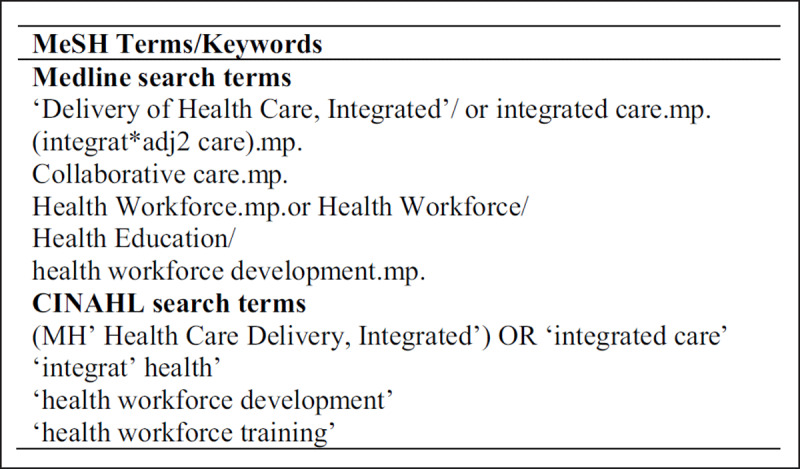
Literature Review Keywords and MeSH Terms. *Notes*: MeSH = Medical subject heading.

All search strategies and databases (see ***Table 1***) were developed with the lead researcher and context experts from the scoping study team, as Levac et al. [17] recommended. A comprehensive literature search was conducted in April 2020, followed by the complete search strategy with all identified search terms in May 2020. The words, truncations and medical subject heading (MeSH) terms used and combined for the PubMed search are shown in ***Figure 1***. The final set of databases was selected for their multi-disciplinary content and inclusion of health and social services. The review only includes studies published after 2013 when the definition of integrated care used by the WHO and adopted for the present study was introduced.

### Stage 3: Study Selection

The search strategy (see ***Table 1***) was refined through ongoing discussions with the research team. From here, empirical and non-empirical articles were included in the final selection of studies and these were analysed to define the extent, range and nature of the material available.

### Stage 4: Charting the Data

***Table 1*** shows the data that was extracted from the selected studies. These fields were chosen to outline the scoping review process.

### Stage 5: Collation, Summary and Reporting Results

Key results included a list of themes and competencies for workforce training on integrated care and a list of identified training models (see ***Table 1***). Thematic analysis was used to identify the themes and findings of the study. These were articulated into tables to make it easier for the reader to interpret [17].

## Results

**Figure 2:**
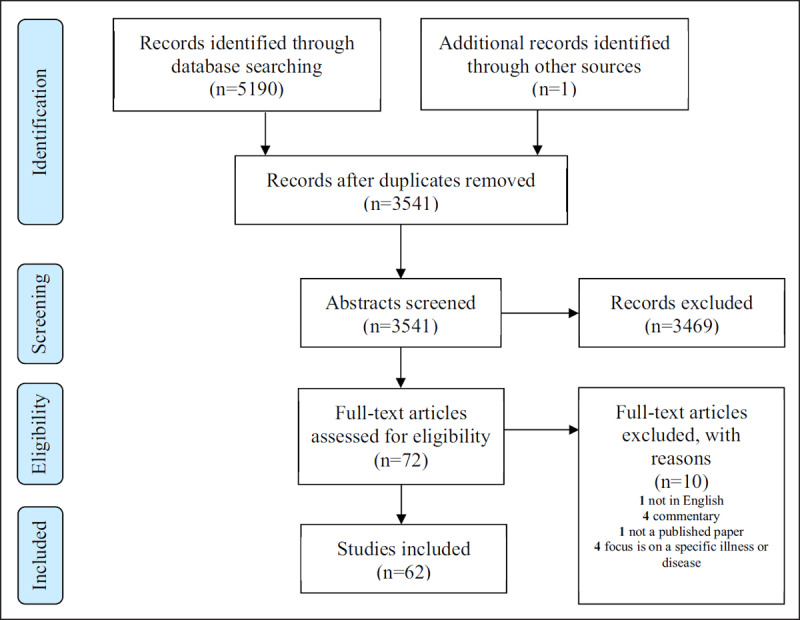
Database Search Results.

The peer-reviewed literature on integrated care and workforce development is sparse, despite international recommendations for preparing the health and social care workforce to work within this model [1,2,3,7]. A total of 5,190 records were first identified in the database search, after which 3,541 records remained following duplicate removal (see ***Figure 2***). The final full-text screening yielded 62 records (see ***Figure 2***).

### Descriptive Characteristics

Most selected studies were empirical and demonstrated systematic collection and analysis of evidence. In contrast, the non-empirical studies included personal reflections, observations, an editorial, a book chapter and systematic reviews (see ***Table 2***). Most studies originated from the United States and Europe (see ***Table 2***). Sixteen of the studies related to training clinicians, such as social workers and psychologists, targeting individuals with mental health or substance abuse conditions.

**Table 2:** Characteristics of the Selected Studies (n = 62).


CHARACTERISTIC	TOTAL N (%)	RELEVANT STUDIES

Type of study		

Empirical	33 (53)	

Non-empirical	29 (47)	

Region		

United States	29 (47)	

Europe	16 (25)	

United Kingdom	7 (11)	

Canada	5 (8)	

International	3 (5)	

Africa	1 (2)	


### Target Workforce

The literature represented workforce groups that generally focused on one or two disciplines. Most common were behavioural health clinicians specialising in mental health conditions, including psychologists, social workers and medical practitioners. Other groups were leaders [20,21], managers [21], primary care professionals [22], expert clinicians [23,24,25,26,27], healthcare students [5], medical graduates [23,28], physicians [29], social service providers in the community [26], health and social care workers or behavioural health providers [29,30,31,32] and educators and academics [23,20]. For example, one study described an integrated care training programme for social workers alongside community social service providers [26].

### Skills and Competencies

Essential skills and competencies were identified and are summarised in ***Table 3***. These include enhancing workforce understanding across the health and social care systems, developing a deeper relationship with patients and their families, patient-centeredness, health promotion, disease prevention and interprofessional education and teamwork.

**Table 3:** Competencies, Themes and References.


	THEMES	SKILLS AND COMPETENCIES	REFERENCES

1	Deeper understanding of our health and social care systems	Enhance workforce understanding of and exposure to alignment of activities across both the health and social care systems	[16,33,11]

2	Deeper understanding of our health and social care systems	Enable workforce attitudes to proactively pursue depth to understand system complexity and how to access services	[15,33,34,11]

3	Deeper understanding of our patients	Skills to construct a comprehensive understanding of individual patients’ complex needs and how these can be met within their surrounding health and social care systems	[34,3,11,13,36]

4	Deeper understanding of our communities	An understanding of how social and cultural factors affect health	[4]

5	Deeper understanding of our communities	Consideration for concerns specific to vulnerable populations and their needs	[34]

6	Deeper understanding of our patients	Skills to actively pursue depth and continuously asking ‘why’ (rather than just ‘what’ or ‘how’) to construct a deep understanding of individual patients (their perceptions, beliefs and psychosocial context) and the system within which they interact	[34,35,11]

7	Deeper understanding of our patients	A holistic understanding of individuals’ health and wellbeing, capabilities, self-management abilities, needs, preferences and the environment in which they find themselves, including recognition that an individual’s situation is dynamic, not static and requires regular monitoring	[31,34,35,36]

8	Deeper understanding of our patients	Skills to establish a longitudinal alliance with the patient and functional relationships with colleagues	[27,28,31,35,11,36]

9	Enhanced understanding of systems and available resources	Extensive integrated knowledge of biopsychosocial aspects of disease, systems of care and social determinants of care	

10	Enhanced understanding of systems and available resources	Understanding how to apply knowledge of the major determinants of health given resources available, relevant health policies and system design within a community	

11	Caregiver involvement	Involvement of and communication with caregivers. An active approach to caregiver wellness, including understanding risk factors, recognising signs of caregiver distress, assessing caregiver needs and referring caregivers to care	[16,34,35,37]

12	Caregiver involvement	Direct provision of psychosocial care to caregivers across a spectrum of needs inclusive of bereavement	[4,34]

13	Enhanced understanding of systems and available resources	Familiarity with local and national resources to support social needs and can connect patients and caregivers to such resources, including community-based partners	[4]

14	Enhanced understanding of systems and available resources	Collaborate with community-based partners to improve patient care. Skill development to collaborate with other health providers outside specialist settings	[4,16,33,34,44,11]

15	Illness prevention	Health promotion and disease prevention, including knowledge of and referral to preventative facilities and local programmes and support for lifestyle interventions	[15,37,39,40,11]

16	Enhanced understanding of systems and available resources	Embrace individuals, communities and services as partners in care	[5,33]

17		A person-focused approach that considers the patient’s presenting problem and other medical issues	[5,13,36]

18		Focuses on the needs of individuals, families and communities to improve their quality of care, health outcomes and wellbeing	

19	Empowering patients	Support patients in their involvement in their care by empowering them with knowledge and skills per their capabilities	[5,11,13]

20		Patient-centred and relationship-centred care	[15,5,35]

21	Interprofessional teamwork	Work effectively as a member of an interprofessional team	[15,5,33,11,41,36]

22		Collaborate with individuals and families to develop a personalised care plan to promote health and wellbeing that incorporates integrative approaches, including lifestyle counselling and mind–body strategies	[15]

23	Empowering patients and communities	Facilitate behaviour change in individuals, families and communities to achieve ways of living that promote health, resilience, wellbeing and disease prevention	[15]

24		Obtain an integrative health history that includes mind–body–spirit, nutrition and use of both conventional and integrative therapies	[15]

24	Role models	Practice self-care	[15]

25		Demonstrate basic knowledge of the major health professions, both integrative and conventional	[15]

26		Demonstrate skills to incorporate integrative healthcare into community settings and the healthcare system at large Value continuous learning, become mentors, teachers and peer learners	[15,36] [33,11]

27	Patient centredness	Patient centredness; understanding and facilitating patients’ pathways through the care system	[15,5,36]

28		Collaborating with other providers; strong communication and collaboration skills and the ability to develop strong working relationships with team members are imperative	[24,5,42,43,44]

29	Health promotion and disease prevention	Community-based health education, health promotion and disease prevention	[15,40,45]

30	Health promotion and disease prevention	Knowledge of how to teach patients self-care strategies to stay healthy and how to incorporate the patient’s strengths and resources within their care plan	[15,37]

31		Understanding individuals’ roles in the integrated healthcare team and the ability to articulate this role to other team members	[24,5,36]


### Models to Support Education and Training

The studies describe several models for education and training, varying by target audience and methods (see ***Table 4***). For example, one study developed a framework for action in the WHO European region, developing competency clusters and a competency consolidation cycle [36]. Another study reported developing a 40-hour online module with course content appropriate for a range of primary care practitioners. The focus was on incorporating these competencies into existing curricula [15]. A further study suggested infusing integrated care content into existing curricula, building foundational knowledge, and developing elective courses to enhance and develop integrated care expertise [24]. The literature also described a 26-item validated tool to measure three areas of integrated care expertise among health and social care professionals: (1) generalism, representing the patient, (2) coaching to empower patients to self-manage their care and (3) population health orientation and prevention [46].

**Table 4:** Models of Training.


	MODEL	RELEVANT STUDIES

1	Scale up existing competencies among all practitioners to deliver more integrated care	[15,30,13,36]

2	Incorporate integrated care concepts organically, so that they are fundamental to delivering care	

3	Create a working environment that values wellness and creates a climate of respect and work-life balance	[14,36]

4	Engage faculty teaching staff who convey joy in their work and provide trainees with education around work-life balance, self-reflection and self-improvement	[14]

5	Embed structures to support collaboration and interprofessional learning among colleagues and professions across services, strengthening multisector relationships; multi-organisation training	[33,47,48,11,36]

6	Incorporate simulation-based scenarios using actors from the local community with lived experiences	[49]

7	Incorporate education and support for caregivers, including prevention of health problems and improving quality of life. For example, implement a weekly meeting for caregivers to discuss topics related to the experiences of the patients’ healthcare and their self-care needs	[37]

8	Allow more time for networking, interprofessional education and opportunities for individual service presentations and diverse attendance, including the social care and voluntary sectors	[47,50,36]

9	Case studies, exercises and simulations are encouraged to allow students to interact with the content in as realistic a venue as possible	[42]

10	Focus on soft skills, such as communication, teamwork and relationship building	[5,34,13,41]

11	Focus on skills to build durable relationships with patients, other professionals and caregivers	[5,34]

12	Focus on self-management promotion and skills, including the use of motivational interviewing techniques	[34]

13	Skills to navigate the health and social care systems and work on individualised care plans and assessments	[30,34,47,13]

14	Ongoing mentorship	[38,51]

15	Workplace training, including interprofessional education, strategies for new staff, such as providing an integrated care manual and shadowing opportunities for the new staff member to be placed with different professionals across sectors and services	[51,36]

16	Workplace training, including team meetings, mutual education about workflow or processes or a review of a problematic shared case	[51,52,41,36]

17	Short courses, such as motivational interviewing	

18	Understanding of primary care providers, including how to interface and refer clients	[14]

19	Interprofessional skill development and education for faculty and a willingness and ability for faculty to evaluate and update curriculum in line with changes within the healthcare environment	[16,53,13,36]

20	Blended learning approaches that use discussions among participants, role play, problem-based learning and case application	[15]

21	Provide opportunities for students and healthcare workers to develop interpersonal and interprofessional strategies to consult, coordinate and collaborate routinely in practice	[5,28,41]

22	Create opportunities and a focus on building relationships and care pathways with organisations in the community	[44,11]

23	Include opportunities for critical thinking and reflective practice and the use of case presentations and role-plays	[16]

24	Create opportunities for all disciplines to train, think, create and seek solutions as a unit	[16,28,36]

25	Create an environment where there is a willingness to think differently about how services are delivered to meet the changing needs and expectations of people using health and social care services	[54]

26	Opportunities for broader and more meaningful engagement across health and social care	[54,57]

27	Incorporate and encourage innovative training and development that spans across health and social care	[54,36]

28	Design clinical practice environments to support and enable continuous learning that benefits not just learners, but also patients, communities and providers	[9]

29	Provide opportunities for participants to gain placement experience engaging in team-based assessments and intervention strategies	[24]


In addition to competency training, an emerging health professional education model was suggested to guide integrated workforce development and expansion [23]. Another study used a Delphi method to explore what skills were needed for doctors in training to practice integrated behavioural health, resulting in a list of 21 competencies [29]. A behavioural science approach was used to implement a behaviour change wheel to upskill health and social care staff to focus on preventative, community-based integrated care [40].

The literature highlighted the importance of knowledge transfer and leadership [47,21,11,36] and suitable learning environments [14]. Students need to be placed within primary healthcare environments to learn the principles of integrated care and develop meaningful, long-term connections with patients. Conversely, implementing and these principles in clinical environments is challenging [14].

### Barriers and Challenges

***Table 5*** summarises barriers and challenges identified in the studies to workforce development in integrated care. These barriers include a lack of understanding of integrated care instead of focusing on siloed health and social care systems and training in acute healthcare systems.

**Table 5:** Barriers/Challenges.


	BARRIER/CHALLENGE	RELEVANT STUDIES

1	Siloed competency domains and traditionally siloed health systems	[18,50,43]

2	Current curricula do not promote the acquisition of experience and skills in the community and integrated care settings	[7]

3	Fragmented, outdated and static curricula	

4	Systems that allow only limited and narrow functional relationships with colleagues	[50]

5	Professional training programmes do not adequately prepare clinicians to work in a collaborative and integrative setting	

6	A small number of professionals may receive training within a short course or generalist training programme, but this represents a limited number of professions who are field-ready after their studies	

7	The general nature of integrated care and learning about other services may not align with the expectations of specialty training	[7,50]

8	A lack of consultant-led integrated services, restricting consultant supervision and workforce development in such services	[7]

9	In many training programmes, students learn the principles of primary care but are then placed in clinical environments where it is challenging to implement and practice those principles	[8]

10	Current curricula for higher medical trainees do not promote the acquisition of experience and skills working across services and within integrated care settings	[7]

11	Emphasis on using standardised clinical pathways and specialists who do not fully understand and are unable to facilitate patients’ pathways through the care system	[29,41,51]

12	Time, budget, organisational and logistic constraints and a lack of access to experts to provide training	[9,10]

13	Training still relies on models that emphasise diagnosis and treatment of acute diseases	

14	Hospital specialists seem unaware of general practice conditions, focusing on disease treatment without considering the daily life of the patient and the existence of comorbidities	[52]

15	A lack of a shared system to facilitate transfer of information across settings and time constraints are major barriers to effective care transitions	[52]

16	Observing patients at different disease stages indirectly affected goal setting	[52]

17	The rigid separation of disciplines at the educational level results in a process that can lead to discontent, animosity, fragmented learning, fragmented practice and, subsequently, fragmented care	[24]

18	Although health and social care staff may value joint working to improve quality of care, interprofessional collaboration did not occur routinely due to organisational limitations	[26]

19	Employees and organisations had limited understanding of integrated care practices	[48]


### Recommendations

Practice recommendations identified from this scoping review are broadly categorised into the following: (1) student selection, (2) faculty selection, (3) curriculum design, (4) workplace, (5) community participation and (6) health system (see Supplemental Table 1).

Good health and social care depend on the workforce overcoming barriers identified in this scoping review and accepting that the biomedical model alone cannot satisfy modern health care [61]. Moreover, health and social services need to be integrated and work effectively together, focusing on preventing rather than curing [62]. Further, the scoping review shows that implementing an integrated information system accessible to all health professionals is central to integrating care in workforce development [60,11,13,36]. The review also found consensus that respect and trust are essential to successful collaboration and that time is required to build and sustain these qualities [60,21,13].

Workforce planning and interprofessional education and practice is essential when implementing a system in integrated care and must be designed around patients and populations, not professions [9,21,11], representing a shift away from silo-based analyses of workforce needs. Instead, different professional groups have flexible, dynamic and overlapping practice areas [6]. Thus, workforce planning should include traditional health professions like nurses and physicians and workers employed in health and social care [9,11,41]. Similarly, Aiello and Mellor [48] recommend collective action that connects local innovation and best practice within consistent national frameworks to meet the aspirations of multi-professional health and care workforce across local systems. Such action requires a joined-up, transformational approach at strategic and operational levels from workforce planners and commissioners to enable integrated health care at scale [48,36].

## Discussion

The literature embraces workforce alignment of activities across health and social systems and settings [8,26] and expands expertise in integrated care education to develop leaders and role models [20,21,23,64,11,36]. Studies also report implementing joint assessments and interprofessional training to overcome interprofessional barriers to a lack of communication and understanding of job roles [30,21,11,36]. Overcoming these barriers will enable participants from both health and social care settings to understand their roles and identify the needs of complex service users [30]. However, the literature does not provide detailed descriptions of how to implement this training. In addition to competency training, one study recommended an emerging health professional education model to guide integrated workforce development and expansion [23]. This model promotes adaptive expertise as a conceptual framework for training healthcare providers to deeply understand patient and system complexity while upholding a patient-centred approach [23]. Adaptive expertise uses more experienced healthcare providers’ extensive knowledge to solve known (routine) and new, complex problems [6]. Implementing an interprofessional education framework [23] will support health and social care providers proactively thinking beyond professional tasks and standardised pathways. Building deeper relationships with patients and more functional relationships with colleagues and other service providers will result in an integrated knowledge of biopsychosocial aspects of disease and systems and social determinants of care [33,11].

The selected studies suggested that training programmes need to incorporate caregiver training, education and support [20], although detailed descriptions of how to implement this training were not provided. Moreover, few studies mentioned patient, carer or community participants actively collaborating to design and deliver the education programmes, which is one of the key principles of integrated care [1].

Only small-scale studies limited to specific health professionals such as physicians, psychologists and social workers were found in this scoping review. Those found were based predominantly in the United States or Europe. Thus, the literature provides no examples from resource-poor countries, international studies or consensus from a range of experts across countries and professions. The selected studies favoured siloed approaches with no studies mentioning other professions such as nursing, allied health and social care. Traditional siloed models no longer provide an appropriate response to patient need. Therefore, we need to find ways to use, prepare and train the more comprehensive health and care workforce to manage an ever-increasing and diverse patient population.

The literature is primarily composed of journal articles presenting opinions along with literature reviews. The selected studies were descriptive but general about the nature of workforce development in integrated care. Descriptions of education and training were predominately aimed at highly qualified and academically trained professionals, especially doctors and social workers. A limited number of studies specifically discussed workforce competencies and education and training models but primarily addressed management and leadership. Education and training need to considerably move up the ladder of priorities if we want to achieve sustainable integrated care in the next generation.

Although a range of competency tools and education frameworks have been developed [36], no studies discuss the implementation and evaluation of these frameworks or measure competency over time. Implementing a regulatory framework for learning environments and organisations will enable workforce changes and integrated care models [20]. The engagement of professional bodies and associations in developing competency frameworks would also help [36]. New leadership, management and professional roles, new working environments and cross-professional and cross-sectoral collaboration are required to execute these changes [20,21,64,36].

### What Would the Perfect Integrated Care Workforce Look Like?

It helps to have a clear understanding of the characteristics of an ideal collaborative practitioner. The results from this scoping review suggest that in the perfect workplace, health and social care providers have the capacity and knowledge to create personalised solutions for people who present with complex issues and follow standardised health pathways and protocols [4,5,6]. These providers understand national and local systems of care [4,33] but are also willing to challenge and negotiate how health care is provided. They work well within and can collaborate with an interprofessional and intersectoral team [4,15,16,21,33,34,38,64,11,41]. They know and understand their community’s needs and have the time and knowledge to teach and role model to patients, families, carers and communities the self-care strategies they require to stay healthy, rather than wait for a disease to develop [15,16,34,37,39,40]. An ideal healthcare provider involves their patients in all aspects of care and can actively incorporate their strengths and resources into their care plan [15,21,27,5,31,64,11]. Focusing on health promotion and disease prevention, [15,40,45] the ideal healthcare provider manages the patients’ health and care rather than disease and cure and empowers patients and their families to stay well. These health and social care providers also value continuous learning and are mentors, teachers and peer-learners.

## Conclusions

This scoping review has highlighted significant gaps in the research to describe and evaluate workforce training and integrated care development. The knowledge gaps cannot be solved effectively by collecting data across the United States and European countries and focusing on similar disciplines. A global plan is needed to understand the leadership requirements, implementation processes, evaluation outcomes and policy levers to create an integrated, people-centred workforce within diverse healthcare systems and sectors. There is an urgent need to develop new academic programmes, competencies and training models, knowledge transfer, and leadership to build a people-centred health workforce and a more integrated healthcare and social care sector approach. Investments are needed in research and implementation studies to foster a greater understanding of the actual content of care required within these new systems. Practice recommendations identified from this scoping review include: (1) student selection, (2) faculty selection, (3) curriculum design, (4) workplace, (5) community participation and (6) health system.

## Additional File

The additional file for this article can be found as follows:

10.5334/ijic.6004.s1 Appendix. Supplemental Table 1 Recommendations for Workforce Development in Integrated Care.
