# Supplementary material for: Workforce Development in Integrated Care: A Scoping Review
Source: Int J Integr Care. 2021 Nov 25;21(4):23. doi: 10.5334/ijic.6004 (PMC8622255; doi:10.5334/ijic.6004)
Supplement: Appendix. — Supplemental Table 1 Recommendations for Workforce Development in Integrated Care. [file ijic-21-4-6004-s1.pdf]

Appendix

Supplemental Table 1 Recommendations for Workforce Development in Integrated Care

	Theme	Recommendation	Relevant Studies
1	Student selection	Medical schools should try innovative approaches to the admissions process and seek applicants with broader capabilities and attributes to diversify their graduates, including students with insight and lived experiences of less privileged and marginalised families and socio-economic problems	[23]
2	Faculty selection	Emphasise creating a learning environment that values wellness and creates a climate of respect and work-life balance	[14]
3		Faculty promotion and success should be based on effective, innovative teaching and mentoring and successes common in primary healthcare, not just research productivity or specialty expertise	[14]
4		Importance should be placed on faculty development (e.g., peer group learning and support) in integrated care models, as the faculty may not have been exposed to this mode of practice during their training	[38,36]
5	Curriculum design	A uniform and well-described terminology related to workforce changes in integrated care interventions should be developed	
6		There should be meaningful, longitudinal connections with patients	[14]
7		Practice environments should reinforce working as a healthcare team	[55,36]
8		Health should be construed not only in terms of physical, mental and social wellbeing but also in terms of the ability to adapt and self-manage, to restore, adapt and cope	[34]
9		Adaptive expertise education programmes should be built to optimise care of patients with comorbid physical and mental illnesses, to increase the clinician’s ability to use effective pathways and manage complexity, ambiguity and uncertainty	[6]
10		Health workforce curricula should include training experiences across community and integrated care settings for all graduates	[55]
11		There is a need to understand patient pathways through the care system	[55,33]
12		Inter-organisational and inter-sectoral multidisciplinary provider education is needed to underpin integrated clinical care	
13		Healthcare workers should be provided with opportunities to focus their expertise on the local contexts where they are working, with a focus on guiding patients throughout their treatment and support them in managing their health	[56]
14		There is a need for interprofessional teamwork that encompasses both face-to-face and virtual teams	
15		A fellowship model should be developed for training general practitioners, which provides a framework to train general practitioners to work in an enhanced manner across primary, urgent and emergency care settings. The model also focuses on developing clinical, academic and leadership skills to influence service	[33]

	Theme	Recommendation	Relevant Studies
		improvement and integration. The model extends understanding of the care pathways and resources available within the community beyond that gained during vocational training and facilitates awareness of community-based care within hospital and urgent care settings	
16		Seek out opportunities to involve students and healthcare practitioners in new and expanding settings [53–55], including opportunities for greater immersion in local community organisations	[55,57,58]
17		Develop a training model that includes clinical opportunities with multiple members of the primary care-based team and didactic/interactive sessions to discuss the implementation of evolving models of treatment	[55,57]
18		Allow opportunities for individual service presentations, networking and diverse attendance, including the social care and voluntary sectors	[50,]
19		Case studies, exercises and simulations should be encouraged to allow students to interact with the content in as realistic a venue as possible	[42]
20		Provide opportunities for students to add value to patient care and health systems, and broader experiences so students can enhance their professional role identity, become change agents and influence care	[59]
21		Provide opportunities for students to gain insight into the lives of the less privileged and marginalised families, and experiences with working with people and families from trauma backgrounds	[23]
22		There is a need to understand the fundamental differences between episodic illness that is identified and cured and chronic conditions that require management across years	
23		Environments should be created where professionals from different background can be trained together, and then be supported in their subsequent work in practice environments that reinforce working as a healthcare team	
24		Provide opportunities for participants to gain placement experience engaging in team-based assessments and intervention strategies	[24]
25		Develop an education model in which interprofessional education shifts away from predominantly focusing on students in the pipeline to designing clinical practice environments to support continuous learning that benefits not just learners, but also patients, populations and providers	[9,36]
26		Expand the students' scope of placement experience to include hard-to-serve populations such as rural settings and working with older people in residential aged care	[53]
27		Include opportunities to learn the skills of case management across multiple practice settings and beyond medical case management, including discharge planning and linking patients to resources and services outside acute care	[24]
28	Workplace	Implement a team orientation, and include specialists and a wide range of staff from various services	
29		Implement a robust information and resource structure	
30		Include training on understanding patient pathways and resources available	[33]
31		Provide an encouraging and supportive environment where staff feel meaningfully supported by a trusted	

Theme	Recommendation	Relevant Studies
	network of peers and teammates	
32	Provide opportunities for new team members to shadow professionals from other sectors or services to learn about and appreciate the contributions of other roles, to build rapport with these services and to better understand the patient experience of care and how team members and services interface	[51]
33	Provide a training manual to highlight the organisation's mission and vision, the value placed on integrated care and what this looks like in terms of workflows and procedures	[51]
34	Provide ongoing team meetings involving all services to enhance rapport among members, create opportunities for sharing different perspectives or approaches to care and provide mutual education, such as workflow, processes or review of a problematic shared case	[51]
35	Provide continuing professional development; for example, hire a consultant to reform the workplace and care delivery, short courses (e.g., motivational interviewing, distance learning, online learning modules and two-day workshops that include faculty who impart key principles, real-life examples, case studies and role-plays)	[41,36]
36	Provide intensive clinical supervision or hire a workplace consultant	[41]
37	Implement training with family medicine practitioners, including mental health and behavioural health teams	
38	Use core competencies on integrated care to shape job descriptions, orientation programmes, supervision, performance reviews for workers, as a resource for educators to shape curricula and training programmes on integrated care	[44]
39	Implement a tiered training framework that scales up existing competencies among all practitioners to deliver more integrated care and incorporates interprofessional education and delivery methods (e.g., simulation, meeting classes, e-learning and the development of clinical champions)	[4]
40	Overcome barriers to sub-specialisation in medicine teams, which creates discomfort in following recommended strategies otherwise critical to understanding patients beyond the scope of a specialised team	[23]
41	Provide a framework for understanding how primary care and behavioural health services can be integrated into routine practice	
42	Build relationships and care pathways with organisations in the community	[16,36]
43	Committed leadership with transparent communication processes and frontline staff members who are willing to take responsibility for communication are crucial when bringing different cultures together	[20,60]
44	Patient care plans, which should reflect patient goals and preferences and referral to social care services, need to be designed in conjunction with health and staff training on the care plans	[60]
45	Develop strategies to help integrate sectors, enabling them to understand each other's roles and engender a perspective that their work was complementary to others. Strategies include knowledge-sharing meetings at which representatives from each setting discuss a sample of patient cases to address incentives, barriers, strengths, weaknesses and opportunities to provide high-quality and well-integrated patient pathways	[60]

	Theme	Recommendation	Relevant Studies
46		Allocate time to shadow colleagues working in other sectors	[60]
47		Managers should consistently share a vision of integration with employees	[60]
48	Consumer participation	Public participation (e.g., open meetings, public workshops, public satisfaction surveys and citizen advisory committees) is important to improve workforce competencies to promote responsiveness to the public's needs, transparency and trust. Actors should be engaged in the topic, exchange ideas, drive coalitions and build a sense of ownership	[61]
49		An individual's self-management abilities are especially relevant in multi-morbidity, as they need to deal simultaneously with multiple problems and providers that may work in different sectors	[34]
50		An individual with multi-morbidity often needs to make choices and set priorities when it is too demanding to address multiple health problems simultaneously. Hence, professionals need to encourage people with multi-morbidity to clarify their personal goals, preferences and priorities	[34]
51		Attention should increasingly shift to the patient's perspective on staffing issues and what is needed according to the patient	
52		The population's unmet needs should be understood when developing integrated services	[62]
53		When creating simulation scenario-based education, the actors' lived experiences as members of the cultural community and as individuals with significant experience with the disease at the focus of the scenario should be considered	[49]
54		Research and evaluation related to integrated care should include patient and family experiences, as integrating care requires ongoing patient involvement to ensure that user needs and expectations are addressed	[60]
55	Health system	Improvements in accreditation requirements and leadership from government agencies are required	[4]
56		Advancing knowledge in workforce changes in integrated care interventions would help decision-makers to design more appropriate integrated care interventions	
57		Practitioners and policymakers should be aware of the emergence of new stakeholders who are assuming more important roles in long-term care, including both for-profit and not-for-profit private enterprises	
58		The implementation of an integrated information system accessible to all health professionals should be a central factor in integrating care	[60,36]
59		Social care and public health representation on boards should be ensured	[54]
60		Education and training should be incorporated into commissioning priorities	[54]
61		Health-related skills, supporting providers and using performance measures of integration should be built into social care	[54]
62		Governments should be aware when interprofessional collaboration exists across healthcare and agencies when considering ongoing funding and models of service delivery	[63]
63		Implement a system in which workforce planning and interprofessional practice and education are designed	[9]

	Theme	Recommendation	Relevant Studies
		around patients and populations and not professions	
64		It should be recognised that different professional groups have flexible, dynamic and overlapping scopes of practice, and planning should include workers employed not only in traditional health professions (e.g., nurses and physicians) but also workers employed in both health and social care	[9,36]
65		Collective action is recommended that connects local innovation and best practice within consistent national frameworks to meet the aspirations of a multi-professional, health and care workforce across local systems	[48]
